# Performance of SS304 Modified by Silver Micro/Nano-Dendrite Coating with Hot-Water Super-Repellency in Simulated PEMFC Cathode Environment

**DOI:** 10.3390/nano12101726

**Published:** 2022-05-18

**Authors:** Junji Xuan, Bingzhi Li, Likun Xu, Zhaoqi Zhang, Yonglei Xin, Lili Xue, Li Li

**Affiliations:** 1 College of Materials Science and Chemical Engineering, Harbin Engineering University, Harbin 150001, China; xuanjunji@gmail.com (J.X.); xuelili@hrbeu.edu.cn (L.X.); lili_heu@hrbeu.edu.cn (L.L.); 2 State Key Laboratory for Marine Corrosion and Protection, Luoyang Ship Material Research Institute, Qingdao 266237, China; lbz11666888@163.com (B.L.); xinyl@sunrui.net (Y.X.)

**Keywords:** bipolar plates, stainless steel, PEMFC, hot-water super-repellency, silver, n-dodecyl mercaptan, electrodeposition, corrosion resistance, conductivity, wettability

## Abstract

In this study, an silver (Ag) plating with micro/nano-dendrite structures is prepared on the 304 stainless steel (SS304) surface by potentiostatic deposition (Ag/SS304). After being modified by n-dodecyl mercaptan (NDM) with the low surface energy, the obtained sample (NDM@Ag/SS304) exhibits stable superhydrophobicity and excellent hot-water repellency. The surface morphology and composition of NDM@Ag/SS304 are analyzed by scanning electron microscope (SEM), X-ray spectrometer (EDS), X-ray diffractometer (XRD), and X-ray photoelectron spectrometer (XPS) characterization. The electrochemical measurements, tests of water contact angle (WCA), and interfacial contact resistance (ICR) are employed to systematically study the performance of the NDM@Ag/SS304 in the simulated cathode environment of proton exchange membrane fuel cell (PEMFC). The results show that the NDM@Ag/SS304 has high corrosion potential (~0.25 V) and low corrosion current density (~4.04 μA/cm^2^); after potentiostatic polarization (0.6 V, 5 h), the NDM@Ag/SS304 also shows high superhydrophobic stability.

## 1. Introduction

Proton exchange membrane fuel cell (PEMFC) is a green and efficient hydrogen power generation device [1]. Bipolar plates, as the core conductive component of PEMFC [2,3], usually work in an environmental condition with weak acidity (pH, 3~6) [4,5] and high temperature of about 80 °C [6]. The severe PEMFC environment requires bipolar plates to possess excellent corrosion resistance [7,8,9], extremely high conductivity [10], and low wettability [11], in order to achieve the efficient, safe, and stable operation of PEMFC as well as the discharge of product water. Stainless steel (SS), especially SS304, has been widely studied as an excellent candidate material for bipolar plates due to its balanced performance [12], low material cost, rapid passivation ability, and outstanding corrosion resistance [13,14,15]. However, the passivation of stainless steel can seriously reduce the conductivity and hydrophobicity of the surface, thus affecting the performance of bipolar plates, and almost all stainless steels need to be addressed by surface modification, e.g., SS310S [16], SS304 [17], SS304L [18], SS316L [19], SS410 [20], SS430 [21], 2205 duplex SS [22]. At present, numerous surface modification studies with various coatings have been carried out on SS304 bipolar plates, such as carbon-based film [23], metal or alloy [24], metal oxide [25], conductive ceramic [17], and conductive composite coating [26]. Nevertheless, most of the research on coating systems only focuses on improving the corrosion resistance or surface conductivity of stainless-steel bipolar plates, and few studies are able to achieve their high surface hydrophobicity. The surface wettability of bipolar plates not only affects the smooth discharge of product water [10], but also influences the corrosion behavior of the surface [27]. High surface wettability can promote the adhesion of corrosive solution and thus easily aggravate local corrosion [11,27]. Furthermore, the wettability of bipolar plates is closely related to the water management of PEMFC [28,29], and good water management can significantly improve the working performance of PEMFC [30]. Therefore, it is of great significance to develop SS304-based bipolar plates with high surface hydrophobicity, but it is far from enough to have high repellency to room temperature water. The working temperature of bipolar plates is as high as 80 °C, and their surface wettability increases significantly in an aqueous solution [31,32]. Therefore, it is urgent to develop bipolar plates with high and stable hot-water repellency.

Silver (Ag) is not easily oxidized, demonstrating excellent stability, conductivity, and corrosion resistance. At room temperature, the resistivity of Ag is only 1.59 × 10^−8^ Ω·m [33], and the standard electrode potential Ag ⇌ Ag^+^ is 0.799 V (vs. SHE) [34]. A large body of research shows that the preparation of Ag coating on stainless-steel bipolar plates can markedly ameliorate the conductivity [35,36] and corrosion resistance [35,36,37]. For instance, Feng et al. [35] prepared the silver-implanted SS316L, of which the interfacial contact resistance (ICR ≈ 150 mΩ cm^2^) under the compression pressure of 150 N/cm^2^ is much lower than that (ICR ≈ 350 mΩ cm^2^) of the bare SS316L. Lin et al. [36] investigated the nitrogen and silver co-alloyed SS316L surface by active screen plasma nitriding technology, and the results showed that the corrosion resistance (i.e., decreased corrosion current density from 0.114 to 0.097 mA/cm^2^ and increased corrosion potential from −460 to −417 mV in 0.5 M H_2_SO_4_ + 2 ppm HF) and conductivity (the ICR is around 20 mΩ cm^2^ at 140 N/cm^2^) of the modified surface were all improved remarkably. Huang et al. [37] developed the electroless silver-coated SS304 bipolar plates, which exhibit corrosion potential as high as 186.38 mV and corrosion current density as low as 5.668 nA/cm^2^ in 0.5 M H_2_SO_4_. In contrast, the corrosion potential and corrosion current density of bare SS304 are −421.1 mV and 990.6 nA/cm^2^, respectively. Besides, the Ag plating can exhibit excellent superhydrophobicity after being modified by low surface energy substances, e.g., n-dodecanoic acid (water contact angle, WCA ≈ 157°) [38] and stearic acid (WCA = 153 ± 3° or 152°) [39,40]. As known, superhydrophobization is an important strategy to improve the hydrophobicity of solid surfaces, and superhydrophobic surfaces usually have micro/nanostructures and low surface energy [41]. Ouyang et al. [42] fabricated an Ag plating with abundant micro/nano-dendrite structures on the Ti substrate by the electrodeposition method, and it could achieve excellent superhydrophobicity (WCA = 158 ± 2°) with n-dodecyl mercaptan (NDM) modification. In practice, the electrodeposition technology is one of the main means to prepare Ag plating, and its thickness, morphology, and properties are easy to control [43]. From this perspective, the preparation of superhydrophobic Ag plating with micro/nanostructures by electrodeposition may be an effective method to improve the performance of SS304 bipolar plates.

In this paper, we designed and prepared the Ag plating with micro/nano-dendritic structures on the SS304 surface (Ag/SS304) via potentiostatic deposition, followed by modifying the Ag plating with NDM (NDM@Ag/SS304). The obtained NDM@Ag/SS304 shows superior superhydrophobicity and high hot-water repellency. The infrared thermal imager was employed to record the heat transfer behavior between the hot droplet and NDM@Ag/SS304 surface, exploring the origin of hot-water repellency of the superhydrophobic Ag plating. The surface morphology and composition of NDM@Ag/SS304 were characterized via scanning electron microscope (SEM), X-ray spectrometer (EDS), X-ray diffractometer (XRD), and X-ray photoelectron spectrometer (XPS), and the corrosion behavior and surface properties of NDM@Ag/SS304 in the simulated PEMFC environment were systematically studied.

## 2. Materials and Methods

### 2.1. Materials

The SS304 used in this experiment was commercially purchased, and its main chemical components (wt.%) are Cr (18.43%), Ni (8.21%), Mn (1.12%), Si (0.44%), C (0.025%), and the balanced Fe. Reagents and solvents, including sulfuric acid (H_2_SO_4_, 98.0%), hydrofluoric acid (HF, 40.0%), ethanol (99.7%), acetone (99.5%), aqueous ammonia (28.0%), n-dodecyl mercaptan (NDM, 98.0%), and silver nitrate (AgNO_3_, 99.8%), were purchased from Sinopharm Chemical Reagent Co., Ltd. and can be used without further purification. The information and content of possible impurities in reagents and solvents can be found in Table S1 (Supplementary Materials). The ultrapure water used was prepared in the laboratory. Before the experiment, SS304 substrates needed to be polished with sandpaper, followed by ultrasonically cleaning with acetone, ethanol, and pure water, and then dried at ambient temperature.

### 2.2. Preparation of Superhydrophobic NDM@Ag/SS304

A three-electrode system, including the counter electrode (SS304, 10 mm × 10 mm × 10 mm), the working electrode (platinum niobium wire), and the reference electrode (saturated calomel electrode, SCE), was used to fabricate the Ag plating on the SS304 (i.e., Ag/SS304) via the potentiostatic deposition (9 V, 2 min) at room temperature, and the composition of electroplating solution (200 mL) was AgNO_3_ (0.1 mol/L) and aqueous ammonia (0.6 mol/L) [42]. After the deposition, the sample was cleaned with pure water, ethanol, and acetone in turn, and dried in a vacuum to obtain Ag/SS304. Then, the Ag/SS304 was fixed in a sealed beaker containing 0.1 mL of NDM solution, and the sample and NDM were not in contact with each other. After heating in an electric oven at 60 °C for 2 h, the superhydrophobic NDM@Ag/SS304 sample was obtained. In this procedure, the sealed beaker was be filled with NDM vapor, which can easily react with the bare Ag plating. The purpose of keeping the NDM solution out of contact with the sample was to prevent a large amount of NDM from being adsorbed or reacting on the surface of Ag, which would seriously affect the conductivity of the solid surface. This is because NDM is a non-conductive organic compound, and the surface conductivity is very important for bipolar plates.

### 2.3. Morphology and Composition

The surface morphology of samples was observed by SEM (ULTRA 55, Oberkochen, Germany). The surface roughness of samples was measured by a three-dimensional laser confocal microscope (LEXT OLS4000, Tokyo, Japan), and the results were the average values of three different positions on the three samples. XPS (250Xi, Waltham, MI, USA) and EDS (X-Max, Abingdon, UK) were used to qualitatively and quantitatively analyze the surface composition of the coating. XRD (D8 ADVANCE, Karlsruhe, Germany) with a Cu Kα (λ = 0.15406 nm) source was used to conduct phase analysis.

### 2.4. Wettability, Infrared Thermography, and Interfacial Contact Resistance Tests

The WCA and sliding angle (SA) of the sample were measured by an automatic surface/interfacial tension meter (Attention Theta, Helsinki, Finland). The composition of the water droplet used in the measurement was the same as that of the simulated solution, and the droplet volume was 3 μL. All WCA and SA values were the averages of three samples, and at least three measurements were made at different positions of each sample. The surface temperature was observed and recorded by an infrared thermal imager (225s, Guangzhou, China). During the recording process, the sample was facing the lens at an inclination of 45°. The interfacial contact resistance (ICR) test referred to the method in the literature [4,32,44,45].

### 2.5. Electrochemical Tests

PARSTAT 2273 electrochemical workstation (USA) was used for all electrochemical tests, and the solution of 1 × 10^−5^ mol/L H_2_SO_4_ and 2 ppm HF was utilized to simulate the PEMFC cathode environment. A three-electrode system, including a working electrode (sample itself), a counter electrode (platinum plated niobium wire), and a reference electrode (saturated calomel electrode, SCE), was applied to carry out the electrochemical test at 80°. Before the electrochemical tests, the samples needed to be immersed in the simulated solution for 2 h to ensure the stability of open circuit potential (OCP). For the potentiodynamic polarization curve tests, the potential range of scanning was −0.3 (vs. OCP) ~ 1.2 V (vs. SCE), and the scanning speed was 2 mV/s. In addition, the potentiostatic polarization curves were obtained after running at the simulated working potential of 0.6 V (vs. SCE) for 5 h.

## 3. Results and Discussion

### 3.1. Surface Morphology and Composition

**Figure 1:**
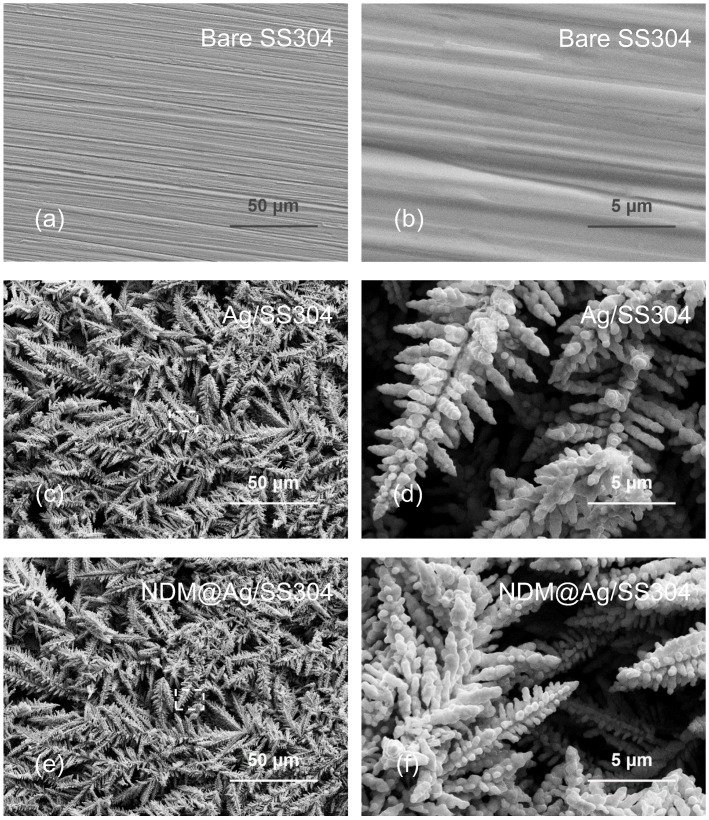
SEM morphology of bare (**a**,**b**) SS304, (**c**,**d**) Ag/SS304, and (**e**,**f**) NDM@Ag/SS304 at (**a**,**c**,**e**) low and (**b**,**d**,**f**) high magnification.

As shown in Figure 1a,b, the bare SS304 at low and high magnification presents a clean and flat micromorphology with slight scratches (caused by sandpaper grinding). After electrodeposition, a large number of micro/nano-dendritic fractal structures are formed on the modified SS304 (Ag/SS304), and all the dendrites are closely staggered, completely covering the whole substrate and forming abundant micro/nanopores, as shown in Figure 1c. Under high magnification (Figure 1d), it is observed that these micro/nano-dendrites, like pine branches, have a main stem with pine needle-shaped side branches. Figure 1e,f displays the surface morphology (at low and high magnification) of electrodeposited SS304 further treated with NDM (NDM@Ag/SS304), and the photos illustrate that the micro/nano-dendritic structures demonstrate no obvious differences before and after NDM modification. The EDS data of the bare SS304, Ag/SS304, and NDM@Ag/SS304 are listed in Table 1, and the corresponding result indicates that the main element of the micro/nano-dendrite structure deposited on the SS304 is Ag. Meanwhile, the composition of Ag on the surface of Ag/SS304 treated with NDM has no significant change compared with that before treatment. Nevertheless, there are sulfur atoms on the surface of NDM@Ag/SS304, but not on the Ag/SS304 surface, which is mainly caused by the NDM modification, because NDM contain sulfhydryl group with sulfur element. In fact, NDM modification does not change the surface morphology of Ag/SS304.

**Figure 2:**
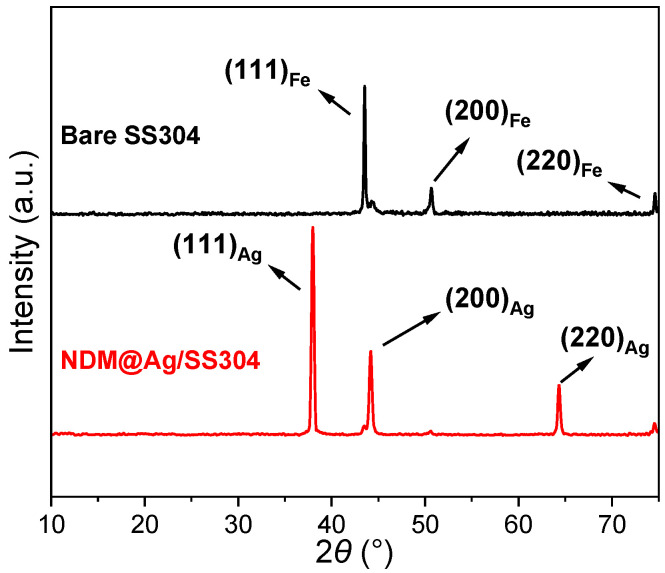
XRD patterns of the bare SS304 and NDM@Ag/SS304.

Figure 2 describes the XRD patterns of the bare SS304 and NDM@Ag/SS304, and it can be found that the bare SS304 only shows the characteristic diffraction peaks of Fe (austenitic structure). For the NDM@Ag/SS304, some new characteristic peaks are observed at 2*θ* = 38.0, 44.2, and 64.3° in the graphs, separately corresponding to the (111), (200), and (220) planes of Ag crystal (ICDD-PDF: 97-018-0878). These results demonstrate that Ag plating is successfully deposited on the bare SS304 surface. Moreover, the weak diffraction peaks from the SS304 in the XRD pattern of the NDM@Ag/SS304 also imply that the electrodeposited Ag plating formed on the SS304 has high crystallinity, compactness, and thickness [42].

**Figure 3:**
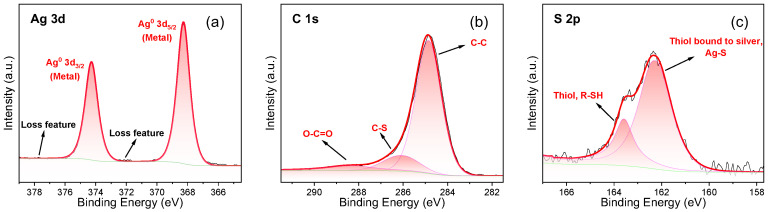
XPS spectra of superhydrophobic Ag plating: (**a**) Ag 3d; (**b**) C 1s; (**c**) S 2p.

XPS analysis was carried out to further confirm the changes of surface composition and chemical state caused by NDM modification (Figure 3). As shown in the XPS spectrum of Ag 3d orbit (Figure 3a), two strong characteristic peaks appear at 368.27 and 374.28 eV due to the spin-orbit splitting. Meanwhile, the characteristic energy loss peaks of metallic Ag can be observed on the left side of these two spin-orbital components, which indicates that these characteristic peaks should be assigned to metallic Ag. However, it is generally believed that the binding energy (BE) peak position of Ag^0^ 3d_5/2_ is about 368.3 eV, and the shift of BE peak to lower BE side is related to the chemical bonding of Ag and other compounds [40,46,47,48,49]. The above inference can be verified by the analysis of the XPS spectra of C 1s and S 2p orbits. As shown in Figure 3b, the characteristic peaks belonging to C-C (284.80 eV) and C-S (286.01 eV) appear in the XPS spectrum of C 1s orbit, which proves the existence of NDM [46]. Moreover, as seen in the XPS spectrum of the S 2p orbit, there are two characteristic peaks at 162.24 and 163.58 eV, which are respectively consistent with the characteristic BE peaks of the coordination bond between NDM and metal (Ag-S) and the thiol group (R-SH) of NDM itself, and the Ag-S peak is much higher than the R-SH peak. The result suggests that a large amount of NDM is combined with metallic Ag by chemical bonding, mainly coordination bonding, while a small amount of NDM is physically adsorbed on the Ag plating surface in its original form [42].

### 3.2. Surface Wettability

**Figure 4:**
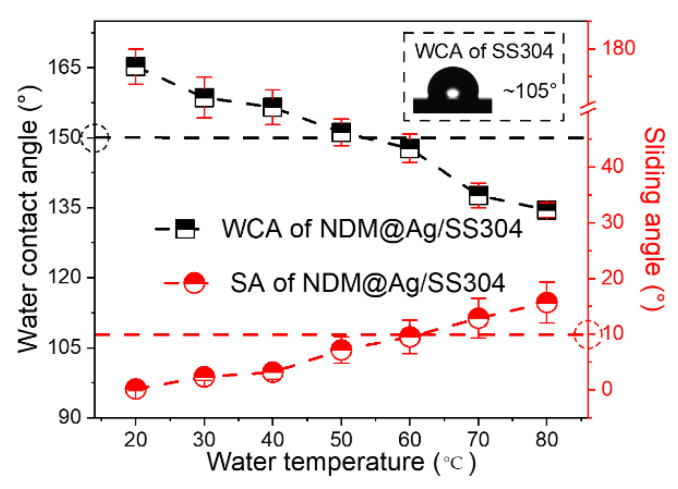
WCAs and SAs of NDM@Ag/SS304 under water droplets at different temperatures.

The pristine SS304 deposited with Ag plating shows superhydrophilicity (i.e., Ag/SS304, WCA < 10°); after NDM modification, the NDM@Ag/SS304 can achieve superhydrophobicity (WCA ≥ 150°, SA ≤ 10°). However, for bipolar plates, it is not enough to realize superhydrophobicity only of room temperature water. If the surface has high hot-water repellency, it is more in line with the practical application requirements in the PEMFC environment (~80 °C). Therefore, the surface wettability of the NDM@Ag/SS304 under water droplets at different temperatures are systematically studied, as shown in Figure 4. Under water droplets below 60 °C, WCAs and SAs of the NDM@Ag/SS304 surface can meet the definition of superhydrophobicity. As for water droplets above 60 °C, the surface can maintain a high WCA (>130°) and low SA (<20°), showing good hot-water repellency. Compared with the bare SS304 (the inset in Figure 4, the WCA under water droplet at room temperature is about 105°), the NDM@Ag/SS304 displays a great improvement in hot-water repellency, even in hot-water super-repellency, which has not been realized by any other bipolar plate research up to now.

### 3.3. Surface Heat Transfer Behavior

Hot-water super-repellency is difficult to achieve, and most superhydrophobic surfaces cannot repel hot water. As is known, the superhydrophobic surface is usually related to the formation of the Cassie-Baxter wetting state, in which much air is trapped between the water and solid interface, forming a stable intermediate air layer. However, when hot water is involved, its evaporation rate is very high and the condensation of hot steam on the cold surface is particularly obvious. All these processes are spontaneous and inevitable, which also increases the possibility that the air layer on the superhydrophobic surface is filled with condensate, thus forming a large number of liquid bridges connecting the solid-liquid interface. Therefore, the temperature difference is usually the most important driving force affecting the wetting of the solid surface by hot water. For example, in 1756, Leidenfrost [50] discovered that the water under normal atmospheric temperature did not wet the hot surfaces. Yu et al. [51] also proved that when the heated solid surface came into contact with hot-water droplets, the smaller the temperature difference between them, the larger the WCA and the smaller the SA of the hot surface, that is, the lower the surface wettability. However, a solid surface can be heated by artificial heating, and it is also possible to be heated by higher temperature objects through heat transfer, but the latter process is usually not evident and easily neglected. If the temperature difference between the hot water and the solid surface remains the constant during the contact, it is very unreasonable to realize hot-water super-repellency, which is because during this process, the hot water will not only evaporate strongly, but also the generated high-temperature steam will condense quickly on the cold surface.

**Figure 5:**
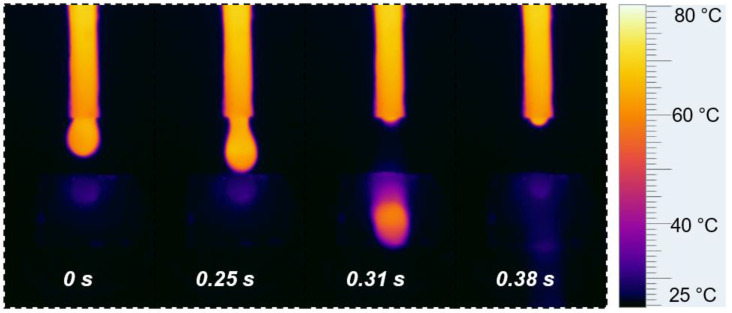
The process of hot-water droplet dripping on the NDM@Ag/SS304 surface that recorded by an infrared thermal imager.

To verify our conjecture, the process of a hot-water droplet contacting the NDM@Ag/SS304 surface was recorded by an infrared thermal imager. As seen in Figure 5, when the hot-water droplet (~80 °C) approaches the surface (room temperature), the weak heat exchange between them has already started; meanwhile, the temperature at the bottom of the droplet decreases slightly, while the temperature at the surface directly below increases slightly. When the droplet leaves the pipette and touches the NDM@Ag/SS304 surface, the temperature of the contact part between them rises rapidly (close to 45 °C). Until the droplet slides down the surface, the temperature of the contacted area on the solid surface is still high (close to 35 °C). During this process, the contact time between the hot-water droplet and the NDM@Ag/SS304 is not more than 0.1 s, demonstrating that the heat transfer process between the hot-water droplet and the NDM@Ag/SS304 is very rapid, which is closely related to the high thermal conductivity of the metal surface. The above research reveals that the heat transfer process between the solid-liquid interface is very significant, during which the thermal conductivity of the solid surface plays a critical role. However, all these factors have not been given enough attention in the past. Exploring the mechanism of hot-water super-repellency of solid surfaces is beneficial to the design of bipolar plate coating, so as to achieve better hydrophobicity in the high-temperature PEMFC environment. Moreover, the research of hot-water super-repellency can also promote the application of related technologies, such as scald prevention, corrosion prevention, oil-water separation, and seawater desalination, and provide a strong theoretical basis to develop more advanced and effective solutions [32,52,53,54].

### 3.4. Corrosion Behavior and Surface Properties of NDM@Ag/SS304 in Simulated PEMFC Cathode Environment

**Figure 6:**
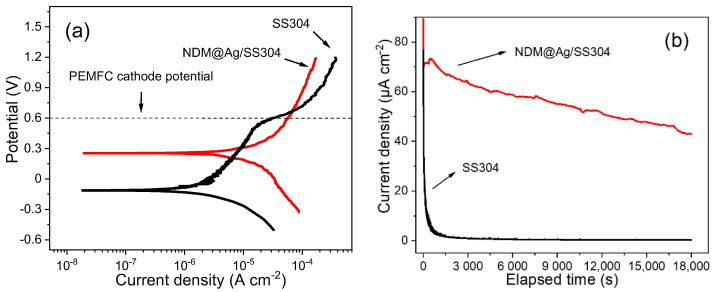
The polarization curves of SS304 and NDM@Ag/SS304 in simulated PEMFC cathode environment: (**a**) potentiodynamic polarization curves, (**b**) potentiostatic polarization curves (0.6 V, 5 h).

Generally, the PEMFC environment can be divided into anode environment and cathode environment. With the development of PEMFC technology, the anode environment is gradually changing, showing high uncertainty. In addition, the corrosion of stainless-steel bipolar plates in the PEMFC cathode environment is usually more serious. To understand the corrosion behavior of the NDM@Ag/SS304 in the simulated PEMFC cathode environment, potentiodynamic and potentiostatic polarization tests were conducted (Figure 6). As shown in Figure 6a, the cathodic polarization curves of the bare SS304 and NDM@Ag/SS304 show similar behaviors; however, the current density of the NDM@Ag/SS304 is slightly higher than that of the bare SS304 at the same cathodic polarization potential, demonstrating that the cathodic reaction on the bare SS304 is enhanced after modification, which may be related to the reduction process of Ag-NDM complexes. However, the anodic polarization behaviors of the bare SS304 and NDM@Ag/SS304 display much difference. With regard to the bare SS304, no obvious active-passive transition process in the anode polarization curve is observed, which indicates that it can be passivated spontaneously in a simulated PEMFC cathode environment. Meanwhile, the current density of the bare SS304 slowly increases in proportion with the increase of polarization potential. In the potential range of 0.0~0.4 V, the bare SS304 shows high polarizability, which is closely associated with the dynamic equilibrium process of the dissolution and growth of the passive film. When the potential exceeds the critical value (breakdown potential, *E*_b_), the current density of the bare SS304 increases rapidly, indicating that the passive film begins to break down. As for the NDM@Ag/SS304, with the increase of anode polarization potential, the current density also increases slowly and proportionally, but the polarization rate and current density are both maintained at a high level all along. The polarization parameters of the potentiodynamic polarization curves of the bare SS304 and NDM@Ag/SS304 are listed in Table 2. It can be seen that the corrosion potential (*E*_corr_) of the NDM@Ag/SS304 is evidently higher than that of the bare SS304, while the corrosion current density (*i*_corr_) of them is similar, indicating that the Ag plating and NDM modification treatment (i.e., NDM@Ag/SS304) can improve the corrosion resistance of the bare SS304.

**Figure 7:**
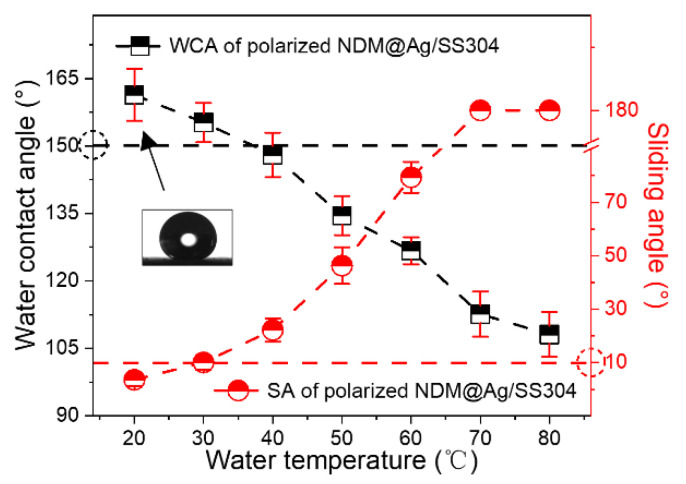
The wettability of NDM@Ag/SS304 after potentiostatic polarization under water droplets at different temperatures in the simulated PEMFC cathode environment.

At the typical cathode working potential of PEMFC (0.6 V, vs. SCE), the bare SS304 and NDM@Ag/SS304 are both in a corrosive state. In addition, the current density (*i*_0.6 V_ vs. _SCE_) of the NDM@Ag/SS304 is slightly higher than that of the SS304 substrate at this polarization potential, which suggests that the corrosion rate of the NDM@Ag/SS304 is marginally faster than that of the SS304 substrate. Nevertheless, at a higher working potential, the NDM@Ag/SS304 demonstrates higher corrosion resistance than the bare SS304. To simulate the long-term operation of NDM@Ag/SS304 in a PEMFC environment, potentiostatic polarization (0.6 V vs. SCE, 5 h) was conducted. As shown in Figure 6b, the current densities of the NDM@Ag/SS304 and the bare SS304 show a trend of continuous decrease, and the eventual stable current density of NDM@Ag/SS304 is about 43.0 μA/cm^2^, which is higher than that of the bare substrate (0.260 μA/cm^2^). Despite that, the polarized surface is still superhydrophobic (as shown in the inset in Figure 7) and also maintains high hot-water repellency (Figure 7). In summary, the NDM@Ag/SS304 shows excellent hydrophobicity in PEMFC working condition, which is greatly improved compared with the bare SS304 in terms of hot-water repellency and stability.

**Figure 8:**
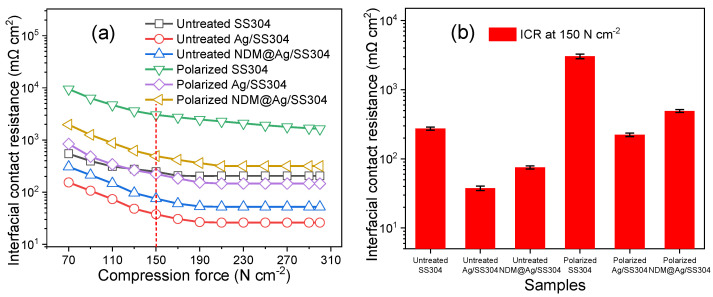
The interfacial contact resistance of SS304, Ag/SS304, and NDM@Ag/SS304 before (untreated) and after potentiostatic polarization (0.6 V, 5 h) in simulated PEMFC cathode environment: (**a**) ICR at various compression forces; (**b**) ICR at 150 N/cm^2^.

Figure 8 shows the ICR of bare SS304, Ag/SS304, and NDM@Ag/SS304, before and after the potentiostatic polarization (0.6 V, 5 h). The ICR values of all samples gradually decrease with the increase of the compression forces (Figure 8a). Under the typical PEMFC stacking compression pressure (i.e., 150 N/cm^2^) of bipolar plates (Figure 8b), the ICR of samples after polarization is greater than that before polarization; however, whether polarized or not, the ICR of Ag/SS304 and NDM@Ag/SS304 is markedly lower than that of the bare SS304. Moreover, the ICR of the NDM@Ag/SS304 surface before and after polarization is marginally higher than that of the corresponding Ag/SS304 surface, which indicates that the modification of NDM with low surface energy has a certain influence on the conductivity of the surface, but the degree of influence is not significant. In brief, the electrodeposited Ag plating can improve the surface conductivity of the bare SS304. At the same time, the modification of micro/nano-dendrite Ag plating with NDM can obtain excellent and stable superhydrophobicity and hot-water repellency, and has little effect on the surface conductivity.

## 4. Conclusions

In conclusion, to enhance the performance of SS304 bipolar plates, this work develops an Ag plating with micro/nano-dendrite structures and outstanding superhydrophobicity (WCA ≥ 150° and SA ≤ 10°) on SS304 by potentiostatic deposition and NDM modification. SEM and EDS results illustrate that the micro/nano-dendrites demonstrate the fractal structure like pine branches, and the NDM modification does not change the morphology of Ag dendrites, but sulfur atoms appear on the treated surface. XPS analysis further reveals that NDM is mainly coordinated on the surface of Ag plating. Moreover, the fabricated NDM@Ag/SS304 has excellent hot-water repellency. The results show that the hot-water super-repellency of NDM@Ag/SS304 is closely related to the high thermal conductivity of the Ag plating, which can reduce the temperature difference between the solid and liquid interface. In the simulated PEMFC cathode environment, NDM@Ag/SS304 displays a high corrosion potential (~0.25 V) and low corrosion current density (~4.04 μA/cm^2^). After potentiostatic polarization (0.6 V, 5 h), the NDM@Ag/SS304 also shows high hydrophobic stability, which is substantially ameliorated compared with the bare SS304 substrate. Moreover, under the compression pressure of 150 N/cm^2^, the ICR of the polarized NDM@Ag/SS304 is much lower than that of the bare SS304. On balance, the superhydrophobic NDM@Ag/SS304 with micro/nano-dendrite structures shows excellent hot-water repellency, remarkable corrosion resistance, and surface conductivity. The developed micro/nano-coating system shows a great performance advantage and latent capacity in the application of bipolar plates for PEMFC.

## Figures and Tables

**Table 1:** Elements and contents (wt.%) of SS304, Ag/SS304, and NDM@Ag/SS304 surfaces obtained by EDS.

Samples	Fe	Cr	Ni	Mn	Si	Ag	C	S
SS304	71.77	18.43	8.21	1.12	0.44	-	0.03	-
Ag/SS304	-	-	-	-	-	99.87	0.13	-
NDM@Ag/SS304	-	-	-	-	-	99.18	0.39	0.43

**Table 2:** Polarization parameters derived from potentiodynamic polarization curves of SS304 and NDM@Ag/SS304, where *E*_corr_ refers to the corrosion potential, *E*_b_ is the breakdown potential, *i*_corr_ denotes the corrosion current density, and *i*_0.6 V_ vs. _SCE_ represent the current density of samples at 0.6 V vs. SCE.

Samples	*E*_corr_ (V vs. SCE)	*E*_b_ (V vs. SCE)	*i*_corr_ (μA/cm^2^)	*i*_0.6 V vs. SCE_ (μA/cm^2^)
SS304	−0.1130	0.5145	3.718	30.77
NDM@Ag/SS304	0.2531	-	4.044	57.16

## Data Availability

The data that support the results of this study are available from the corresponding author upon reasonable request.

## Supplementary Materials

The following supporting information can be downloaded at: https://www.mdpi.com/article/10.3390/nano12101726/s1, Table S1: The information and content of possible impurities in reagents and solvents, the horizontal line (-) in the table represents unknown.
