# Supplementary material for: Performance of SS304 Modified by Silver Micro/Nano-Dendrite Coating with Hot-Water Super-Repellency in Simulated PEMFC Cathode Environment
Source: Nanomaterials (Basel). 2022 May 18;12(10):1726. doi: 10.3390/nano12101726 (PMC9145656; doi:10.3390/nano12101726)
Supplement: Supplementary file 1 [file nanomaterials-12-01726-s001.zip › nanomaterials-1731901-supplementary.pdf]

nanomaterials

Supplementary Materials

Performance of SS304 Modified by Silver Micro/Nano-Dendrite Coating with Hot-Water Super-Repellency in Simulated PEMFC Cathode Environment

Junji Xuan [1,2], Bingzhi Li [2], Likun Xu [1,2,*], Zhaoqi Zhang [2,*], Yonglei Xin [2], Lili Xue [1] and Li Li [1]

1 College of Materials Science and Chemical Engineering, Harbin Engineering University, Harbin 150001, China; xuanjunji@gmail.com (J.X.); xuelili@hrbeu.edu.cn (L.X.); lili_heu@hrbeu.edu.cn (L.L.)
2 State Key Laboratory for Marine Corrosion and Protection, Luoyang Ship Material Research Institute, Qingdao 266237, China; lbz11666888@163.com (B.L.); xinyl@sunrui.net (Y.X.)
* Correspondence: xulk@sunrui.net (L.X.); zhangzhaoqi527@163.com (Z.Z.)

Table S1. The information and content of possible impurities in reagents and solvents, the horizontal line (-) in the table represents unknown.

Impurities	H_2SO_4	HF	Ethanol	Acetone	NH_4OH	n-Dodecyl mercaptan	$AgNO_3$
Burning/evaporation residue	≤ 0.001	≤ 0.002	≤ 0.001	-	≤ 0.002	-	-
Chloride (Cl^-)	≤ 0.00003	≤ 0.001	-	-	≤ 0.00005	-	≤ 0.001
Nitrate (NO_3^-)	≤ 0.00005	-	-	-	-	-	-
Sulfate (SO_4^{2-})	≤ 0.002	≤ 0.002	-	-	≤ 0.0002	-	≤ 0.004
Phosphate (PO_4^{3-})	-	≤ 0.0002	-	-	≤ 0.0001	-	-
Fluosilicate (SiF_6^{2-})	-	≤ 0.04	-	-	-	-	-
Carbonate (CO_3^{2-})	-	-	-	-	≤ 0.001	-	-
Sulfide (S^{2-})	-	-	-	-	≤ 0.00002	-	-
Amine salt (NH_4^+)	≤ 0.0002	-	-	-	-	-	-
Na	-	-	-	-	≤ 0.0005	-	-
K	-	-	-	-	≤ 0.0001	-	-
Mg	-	-	-	-	≤ 0.0001	-	-
Ca	-	-	-	-	≤ 0.0001	-	-
Fe	≤ 0.00005	≤ 0.0001	-	-	≤ 0.00002	-	≤ 0.0002
Cu	≤ 0.00001	-	-	-	≤ 0.00001	-	≤ 0.0007
As	≤ 0.000003	-	-	-		-	-
Pb	≤ 0.00001	≤ 0.0005	-	-	≤ 0.00005	-	≤ 0.0007
Potassium permanganate-reducing (SO_2)	≤ 0.0005	-	-	-	-	-	-
Potassium permanganate-reducing (O)	-	-	≤ 0.00025	-	≤ 0.0008	-	-
Water	-	-	≤ 0.3	≤ 0.3	-	-	-
Ethanol	-	-	-	≤ 0.05	-	-	-
Methanol	-	-	≤ 0.05	≤ 0.05	-	-	-
Isopropyl alcohol	-	-	≤ 0.01	-	-	-	-
